# Supplementary material for: Active site specificity profiling datasets of matrix metalloproteinases (MMPs) 1, 2, 3, 7, 8, 9, 12, 13 and 14
Source: Data Brief. 2016 Feb 22;7:299–310. doi: 10.1016/j.dib.2016.02.036 (PMC4777984; doi:10.1016/j.dib.2016.02.036)
Supplement: Supplementary file 10 — Supplementary material [file mmc10.zip › WebPICS_hMMP8_G_1%/P3.html]

 

PICS results


|  |  |
| --- | --- |
| **P3\_A**  24 in 120 sites   20.0 %    effects > 10 perc. pnts.  (vice-versa in brackets)  P1\_A: 14.2 (26.2) |  |
| **P3\_H**  5 in 120 sites   4.2 %    effects > 10 perc. pnts.  (vice-versa in brackets)  P1\_P: 52.5 (29.1)   P1prime\_F: 54.2 (38.7)   P2\_W: 56.7 (70.8)   P2prime\_Q: 50.0 (20.8)   P3prime\_V: 51.7 (25.8) |  |
| **P3\_I**  9 in 120 sites   7.5 %    effects > 10 perc. pnts.  (vice-versa in brackets)  P2\_L: 31.1 (17.5)   P2prime\_H: 18.0 (32.5)   P2prime\_R: 15.5 (17.5)   P3prime\_A: 45.0 (15.6) |  |
| **P3\_P**  28 in 120 sites   23.3 %    effects > 10 perc. pnts.  (vice-versa in brackets)  P1\_N: -15.6 (-19.0)   P1prime\_L: -37.2 (-21.3)   P1prime\_Q: 18.6 (43.4)   P1prime\_V: 15.0 (35.0)   P2\_K: 12.2 (31.2) |  |
| **P3\_V**  13 in 120 sites   10.8 %    effects > 10 perc. pnts.  (vice-versa in brackets)  P2\_A: 11.4 (10.6)   P2\_N: 10.4 (22.5)   P2prime\_Q: 13.1 (14.2) |  |
